# Supplementary material for: Mitochondrial DNA and Electron Transport Chain Protein Levels Are Altered in Peripheral Nerve Tissues from Donors with HIV Sensory Neuropathy: A Pilot Study
Source: Int J Mol Sci. 2024 Apr 26;25(9):4732. doi: 10.3390/ijms25094732 (PMC11083172; doi:10.3390/ijms25094732)
Supplement: Supplementary file 1 [file ijms-25-04732-s001.zip › Supplementary Data S1.pdf]

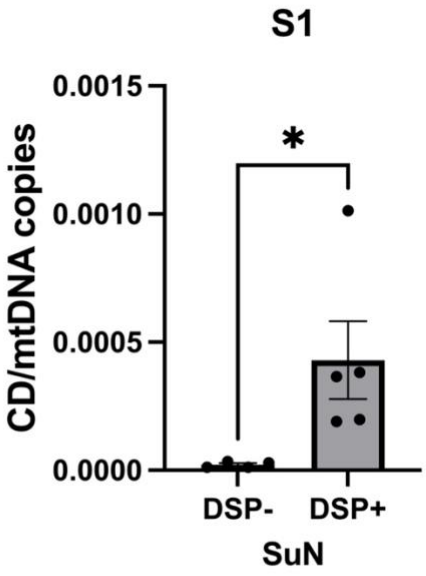

S1: mtDNA common deletion (CD) RA (relative abundance) in SuN (sural nerve) of PWH (people with HIV) with and without Distal sensory polyneuropathy (DSP). Removal of an individual with a markedly higher level of mtDNA common deletion RA (approximately 100 times more) compared to other mtDNA common deletion RA levels from DSP- group resulted in significantly increased mtDNA common deletion in the SuN of DSP+ compared to DSP− decedents.
