# Supplementary material for: Mitochondrial DNA and Electron Transport Chain Protein Levels Are Altered in Peripheral Nerve Tissues from Donors with HIV Sensory Neuropathy: A Pilot Study
Source: Int J Mol Sci. 2024 Apr 26;25(9):4732. doi: 10.3390/ijms25094732 (PMC11083172; doi:10.3390/ijms25094732)
Supplement: Supplementary file 1 [file ijms-25-04732-s001.zip › Supplementary Data S2.pdf]

MFN2:

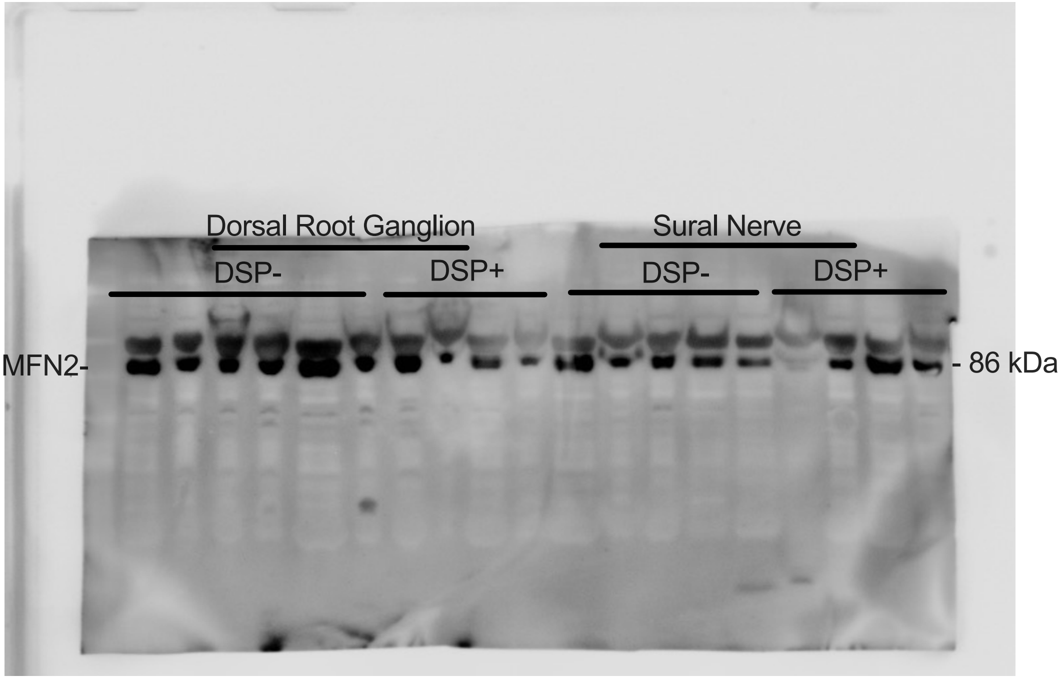
Dorsal Root Ganglion
Sural Nerve
DSP-
DSP+
DSP-
DSP+
MFN2-
- 86 kDa

MFN1:

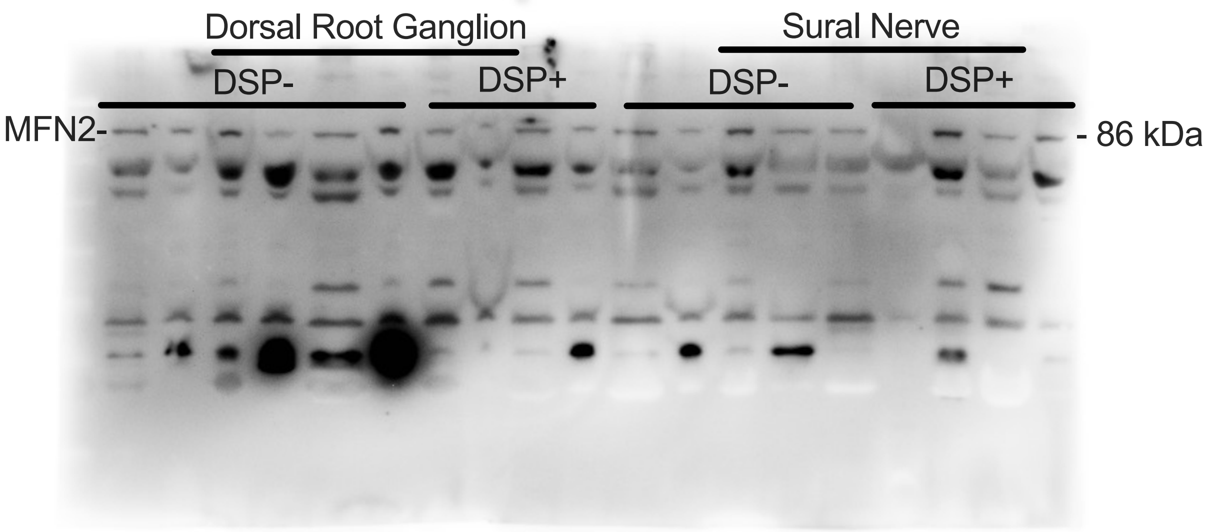
Dorsal Root Ganglion
Sural Nerve
DSP-
DSP+
DSP-
DSP+
MFN2-
- 86 kDa

DRP1:

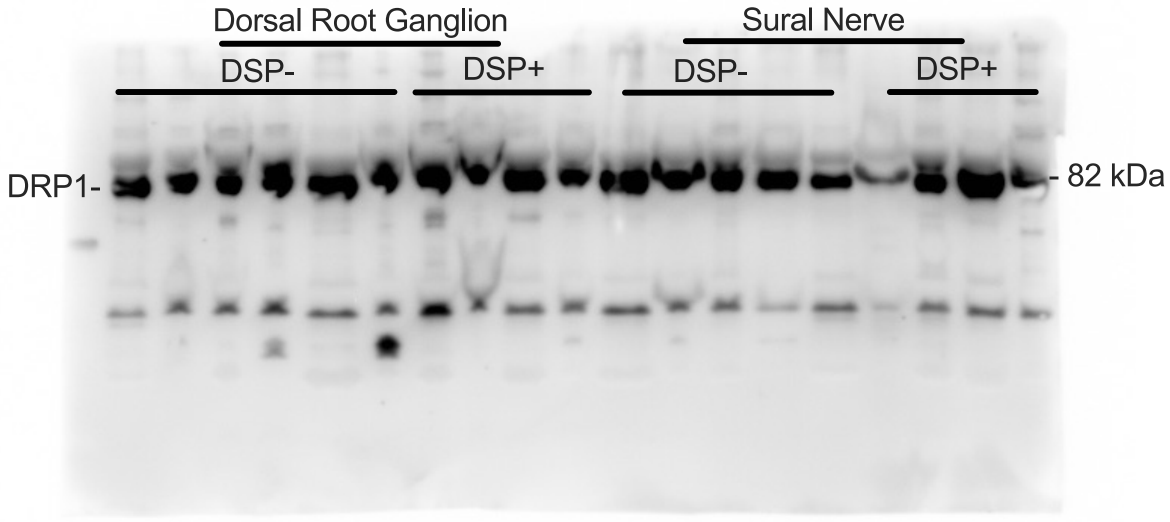
Dorsal Root Ganglion
Sural Nerve
DSP-
DSP+
DSP-
DSP+
DRP1-
- 82 kDa

ACTB (a):

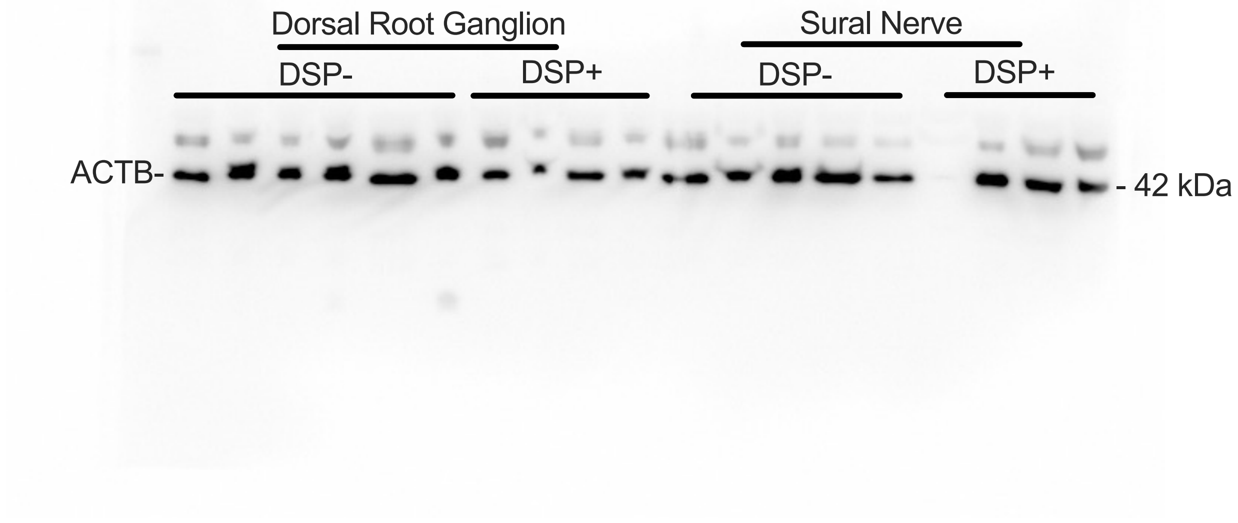
Dorsal Root Ganglion
Sural Nerve
DSP-
DSP+
DSP-
DSP+
ACTB-
- 42 kDa

pDRP1:

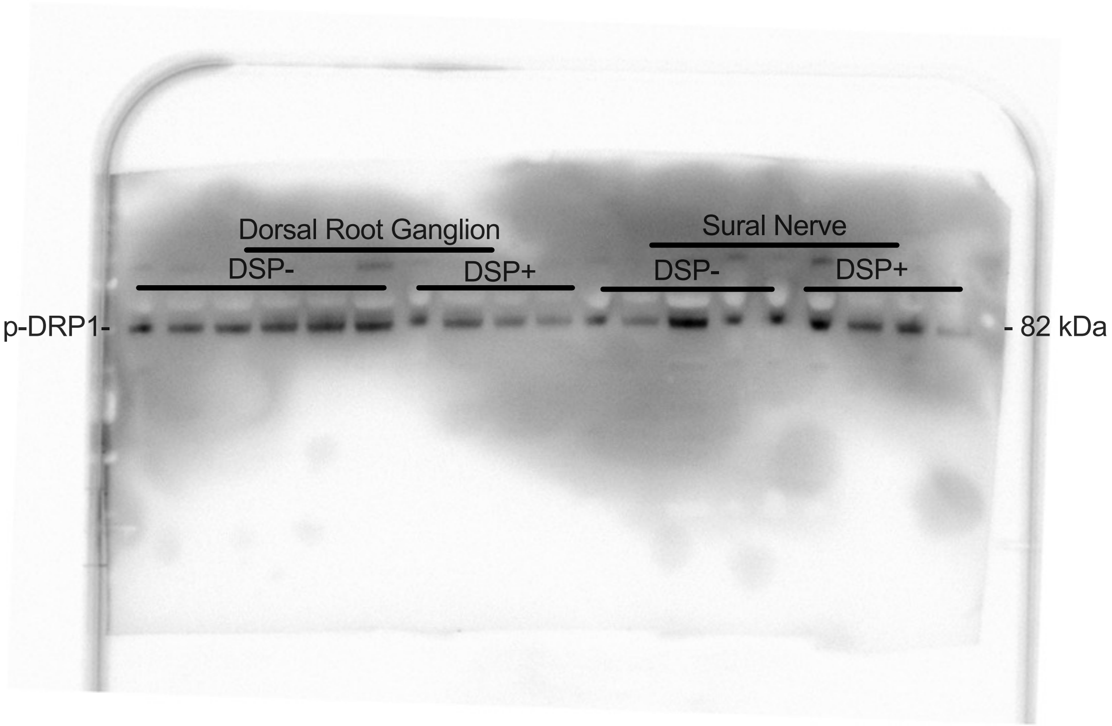
Dorsal Root Ganglion
Sural Nerve
DSP-
DSP+
DSP-
DSP+
p-DRP1-
- 82 kDa

ACTB (e):

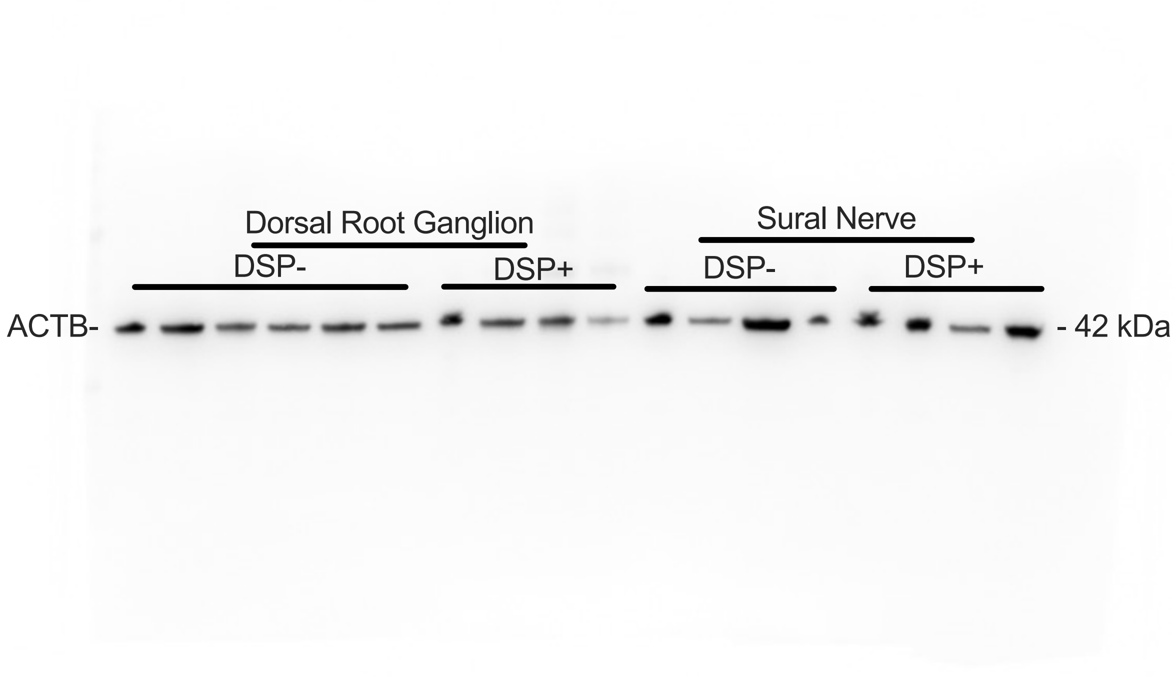
Dorsal Root Ganglion
Sural Nerve
DSP-
DSP+
DSP-
DSP+
ACTB-
- 42 kDa

ETC:

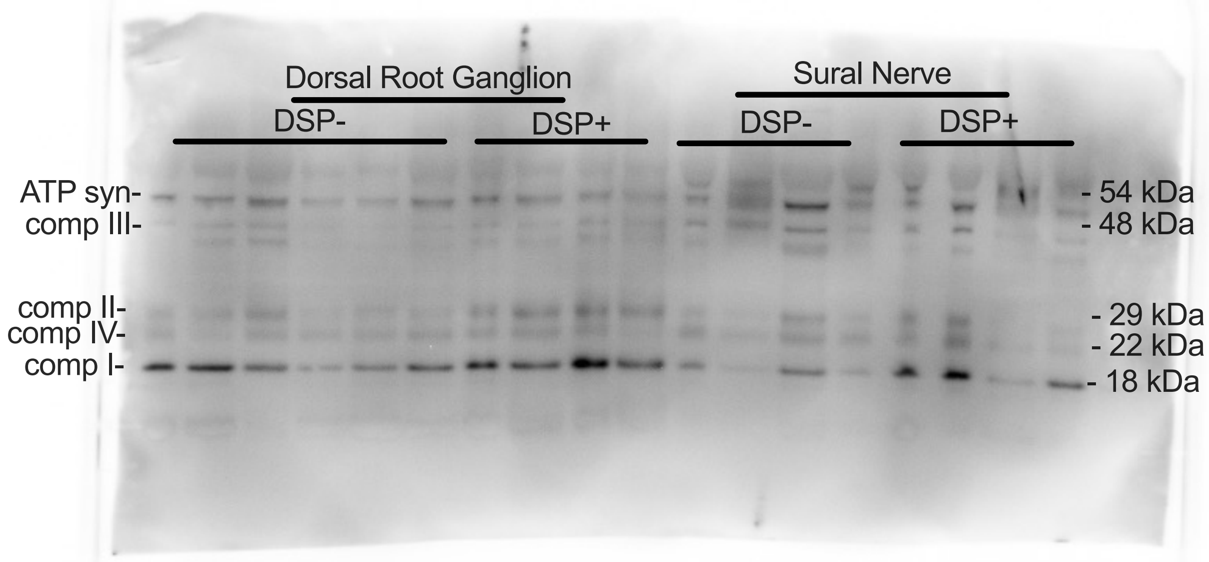
Dorsal Root Ganglion
Sural Nerve
DSP-
DSP+
DSP-
DSP+
ATP syn-
comp III-
comp II-
comp IV-
comp I-
- 54 kDa
- 48 kDa
- 29 kDa
- 22 kDa
- 18 kDa
